# Implementing a layperson post-crash first aid training programme in Tanzania: a qualitative study of stakeholder perspectives

**DOI:** 10.1186/s12889-020-08692-8

**Published:** 2020-05-24

**Authors:** Menti L. Ndile, Britt-Inger Saveman, Anne H. Outwater, Dickson A. Mkoka, Susann Backteman-Erlanson

**Affiliations:** 1 Department of Clinical Nursing, Muhimbili University of Health and Allied Sciences (MUHAS), P.O BOX 65001 Dar es Salaam, Tanzania; 2 Department of Nursing, Umeå University, Umeå, Sweden; 3 Department of Community Nursing, MUHAS, Dar es Salaam, Tanzania

**Keywords:** Post-crash care, Training, Facilitators and barriers, Stakeholder perspective

## Abstract

**Background:**

In low and middle-income countries (LMICs), laypersons play a significant role in providing initial care to injured victims of traffic accidents. Post-crash first aid (PFA) training programmes for laypersons have become an important response to addressing knowledge and skills gaps in pre-hospital care. However, little is known about factors influencing effective implementation of such programmes from stakeholders’ point of view. Therefore, this study aimed to explore views of stakeholders on potential factors that may facilitate or hinder successful implementation of a PFA training programme for lay persons.

**Methods:**

Twelve semi-structured qualitative interviews with leaders at a traffic police department and leaders of an association of city bus drivers, taxi drivers and motorcycle taxis in Tanzania were conducted. Interviews were audio-recorded and transcribed verbatim. A thematic analysis approach was used to identify themes and sub-themes.

**Results:**

Three themes pertaining to implementation of a PFA training programme were identified: Motivation for engaging in training, Constrains for engaging in training and Training processes. They consisted of a total of six sub-themes: “perceived benefits of first aid training” and “availability of incentives” were considered as facilitators to PFA training. “Availability of time to attend training” and “accessibility of training” were reported as a potential barriers to successful training. Finally, they felt that “methods of training delivery” and “availability of first aid training materials and equipment” could either facilitate or impede delivery of PFA training.

**Conclusion:**

This study highlights potential facilitators and barriers to implementing a PFA training programme for lay persons from the perspectives of leaders from police department and associations of city bus drivers, taxi drivers, and motorcycle taxis. This may be useful information for other stakeholders, and may enable government-level leaders and persons higher up in the health service hierarchy to take action to meet WHO recommendations for emergency pre-hospital care.

## Background

About half of all deaths of injured victims of road crashes occur in a pre-hospital environment [1]. Although this is a significant problem, many low and middle-income countries (LMICs) either lack or have inadequate emergency pre-hospital care systems. In these contexts, laypersons play a significant role in providing initial assistance to injured victims of traffic accidents.

Recently, more attention and resources in development of emergency care in LMICs have been focused on in-hospital emergency care; however, emergency care can only be effective and efficient when pre-hospital and in-hospital care systems co-exist [2]. Lack of skilled human resources for emergency pre-hospital care, among other things, contributes to poor post-crash care management of road traffic-injured victims [3]. The majority of people with road traffic injuries (RTIs) in LMICs are taken to emergency care facilities by medically untrained laypersons such as police officers, drivers, relatives, and bystanders [4].

The World Health Organization (WHO) recommends that in limited-resource countries, laypersons should be empowered with basic knowledge and skills for emergency pre-hospital care [2]. Based on this recommendation, a growing number of research studies have been undertaken with the focus on building capacity of laypersons to provide simple first aid interventions [5]. A study conducted in Iraq showed that in case of long pre-hospital transit times, delegating life-saving skills to paramedics and lay persons is a key factor for efficient pre-hospital trauma systems in limited-resource settings [6]. A systematic review describing implementation of first aid programmes indicated that first aid administered at the scene of injury by trained laypersons reduces trauma mortality [5]. Additionally, the literature shows that implementing layperson first aid training programmes is a practical and effective first step towards developing a formal emergency system [7] including pre-hospital care.

In Tanzania, currently there is no national plan for training laypersons. However, the government supports institutions and organization to conduct such trainings. Laypersons’ first aid training are highly needed considering that road traffic injuries and deaths are significant [8], and there is low knowledge and skills of post-crash first aid (PFA). For example, a study conducted among traffic police officers in the country showed that fewer than 10% knew how to manage an airway or position RTI victims [9].

Considering that successful implementation of lay person first aid training programmes can be influenced by a number of contextual factors, no documented studies in Tanzania have assessed the aspect from a stakeholder’s point of view. Therefore, this study aimed to explore views of stakeholders in the public transport sector in Tanzania on potential factors that may facilitate or hinder successful implementation of a layperson PFA training programme.

## Methods

### Study design and setting

An inductive thematic analysis qualitative approach using semi-structured interviews was applied in this study which is part of a larger research project on Injury Prevention and Care in Tanzania (INPACT). The study was conducted in Dar es Salaam region which includes Dar es Salaam, the main commercial city of Tanzania, with an estimated population of more than 5.7 million [10].

### Sample and recruitment

There are several associations and departments involved in road sector, for the purpose of this study drivers’ associations and traffic police department were selected as they closely work together on road safety matters. Twelve leaders from drivers’ associations and traffic police department were purposively selected based on administrative positions they held, for example chairman, secretary and leaders of welfare committees in respective associations and leaders responsible for human resource training in the traffic police department. The participants were chosen as themselves being potential recipient of training programme as well as representing interests of their fellow workers.

The participants were individually invited for a face-to-face interview at an agreed location between February 2018 and January 2019. Written informed consent was secured prior to the interviews. The number of participants were as follows; from the traffic police department (*n* = 2), the city bus drivers’ association (*n* = 3), the taxi drivers’ association (*n* = 4), and the motorcycle taxis (*n* = 3). All participants were male except for one female traffic officer. Their age ranged from 35 to 55 years with varying leadership experiences in their respective positions.

### Data collection

An interview guide was tested on and evaluated by two participants who were not included in the main study. No major changes were made before applying the interview guide to the main study. Individual interviews were then conducted by the first author (M.L.N.) using a semi-structured interview guide which was comprised of five research questions (See Additional file 1). The main questions were: i) Tell me your views concerning current situation in the provision of first aid to road injured victims in the country. ii) Are there any potential factors that may enable implementation of first aid training programme to non-medical persons? Please tell me. iii) Tell me your views regarding potential factors that may hinder implementation of first aid training programme to non-medical persons. Probing questions were asked to gain more understanding of important issues raised during the interviews.

The interviews were conducted in Swahili, lasted for an average of 45 min, and were audio-taped. Prior to data analysis, all interviews were transcribed verbatim to a MS Word document in the original language. Two interviews were further translated into English so that the co-authors not fluent in Swahili could follow the analysis process throughout.

### Data analysis

An inductive thematic analysis was chosen as it is a theoretically flexible approach to analysing qualitative data without being influenced by a pre-existing coding frame [11]. The first author (M.L.N.) firstly went through the interview texts to gain familiarity with the content; thereafter, texts were imported to OpenCode software, version 4.03, for organizing and coding data [12]. Initial coding involved identifying and naming meaning units with codes. A coding frame was developed based on data from the first two interviews; thereafter, M.L.N. used the coding frame to code the remaining interviews. The coding frame was only intended as a guide during the subsequent coding process. All codes from Swahili transcripts were translated into English during analysis process.

Two of the co-authors (B-I.S. and S.B-E.) reviewed the coding process. Disagreement on assigning some codes led to further discussion, until alternative codes were generated and consensus was reached. In the process, some codes were redefined and others merged. Codes were subsequently aggregated into themes. Furthermore themes were translated back to Swahili by the first author and compared with coded extracts from original transcripts in order to determine their coherence. A co-author (D.A.M) who is fluent in both Swahili and English verified the process. Finally themes and sub-themes characterizing facilitation of or barriers to implementation of PFA training were decided on by all authors.

## Results

Three themes pertaining to the implementation of PFA training programme, from the stakeholders’ perspective, were identified: Motivation for engaging in training, Constrains for engaging in training and Training processes. Within the themes, six sub-themes characterizing facilitation of and barriers to PFA training implementation were further identified (Table 1).

**Table 1:** Themes, sub-themes and examples of quotes

Themes	Sub-themes	Example of quotes
Motivation for engaging in training	Perceived benefits of first aid training	*“This training will build our skills so we can be able to do something. Actually, I have never received such training in my life, so it will be good to be [there] in person.”*
	Availability of incentives	*“You may invite 100 people to attend the training but without money to cover the expenses and time, many will not come.”*
Constrains for engaging in training	Availability of time to attend training	*“You may expect many people to come (for the training) but you find only few come; this is because most of them have other important activities to attend to”*
	Accessibility of training	*“People are really busy … so to travel somewhere far to attend training may be a big challenge, certainly for me and others”*
Training processes	Methods of training delivery	*“Actually it will be very important if we actively practise like in a real situation, it will be difficult to forget … and it is more meaningful.”*
	Availability of first aid training materials and equipment	*“If you train us with sophisticated equipment it is good but may not be available in real life. Something that is available locally may be more useful.”*

### Motivation for engaging in training

The first theme describes personal issues the stakeholders considered as important in the facilitation of first aid training implementation. Within this theme, two sub-themes were identified.

#### Perceived benefits of first aid training

The stakeholders viewed the training to have a significant positive impact both for the participants and for the road accident victims. They described that participation in the training would enable their fellow workers to acquire first aid skills, and therefore that they would be in a position to provide proper care to injured victims. Furthermore, they thought that the survival outcome of injured victims would improve significantly if they would be able to get skilled help before they reached the hospital. “*... personally, the skills will enable us to provide proper help to our families and people around us when they get injured*” (Participant 9).

However, for the training to have a meaningful effect, stakeholders suggested that there should be massive training involving as many potential first responders as possible.

The stakeholders also commented that having first aid training might boost the confidence of their fellow workers to care for injured victims: not only would they know what to do but they would also be able to use first aid materials and equipment correctly. “*Therefore this [training] is a good thing and will help our colleagues to be confident to help [injured victims] …*” (Participant 7).

Furthermore, the stakeholders, particularly those from the drivers’ associations, described that the training would likely raise the respect of the public for drivers because they would be seen as people who could make a difference in others’ lives. “*When helping someone and people see that you [have] saved [a] life, people may respect you and even compare you to a doctor!*” (Participant 9).

#### Availability of incentives

There were mixed opinions regarding the type of incentive that could motivate drivers and the police to actively participate in first aid training. The stakeholders of the drivers’ associations stated that financial incentives such as reimbursement for the time spent on training were important to motivate their fellow workers to participate in the training. “*Many drivers don’t get salaries, so if you take them to the training even for 3 hours without compensating them for their time … most of them will not pay attention to the lessons and may leave immediately*” (Participant 6).

On the other hand, stakeholders of the police explained that many fellow police officers would be more likely to participate in the training if, upon completion of the training, they would get a certificate of course attendance/competency which is recognized by the police force as a relevant document for job promotion*.* “*If the training is prepared in such a way [that] we also get [a] certificate that is recognized for promotion … many [police] will be motivated to take part.*” (Participant 1).

### Constrains for engaging in training

The second theme revolved around issues the stakeholders considered as work place constrains in first aid training implementation. Within this theme, two sub-themes were identified.

#### Availability of time to attend training

Stakeholders noted that attending training would be challenging for their colleagues because the training schedule may not be compatible with their work schedule. This was a main concern expressed by leaders of bus and taxi drivers’ associations. “*Honestly, commuter drivers have very limited time...so when planning for training, the timing of the training is very important.*” (Participant 4). To best address this concern, they suggested that training be conducted during the afternoon hours when they were less busy as they had fewer customers. By contrast, the stakeholders for the police officers, who were in formal employment, did not report time for training as a problem.

#### Accessibility to training

Several issues were raised by stakeholders concerning accessibility to the place of training. One of the issue was the distance. Stakeholders described that conducting training near their workplace could make training more reachable and hence increase attendance and time spend on the training contrary if the training is to be conducted faraway. “*Attending training faraway may be difficulty because of time … to avoid that, training can be done near our worksites; this will ensure good participation.”* (Participant 3).

Another issue raised concerning accessibility to the training was related to cost implication if the training was to take place far from worksites. All stakeholders except from police expressed that their colleagues could find it difficult to pay for their fare or fuel to get to the place of training unless they are helped to do so. “*… it will be costly if this training is done far away and I’m afraid people may not be interested unless you pay for their travelling expenses”* (Participant 6)*.*

### Training processes

Two sub-themes were identified under the theme of Training processes.

#### Methods of training delivery

The stakeholders expressed that practical-oriented training was more desirable because it would enable their fellow workers to acquire appropriate first aid skills needed when providing first aid care in the real situation. According to stakeholders from the police department, they are required, as part of their job description, to attend first aid training. However, the training they receive is mainly theoretical, and therefore, when they finish the training they are unable to help in an accident because they lack skills. “*Usually we have some first aid training just in theory … no practical training, so if we have that opportunity [i.e. practical training], that will be very helpful.*” (Participant 1). The same sentiment was shared by leaders of the drivers’ associations who commented that first aid, if taught at all in driving schools, is usually taught as a theoretical subject.

The stakeholders felt that frequent refresher training on first aid should be available. There were mixed views on the number of refresher training sessions; some suggested that the refresher training should be done every 3 months while others suggested once a year. More frequent training was considered as an important facilitator to programme implementation and sustainability because it would mean that the knowledge and skills gained would be maintained over time, as well as updated: “*It’s important that this training is repetitive so that things stick in our heads … it’s, like, today you forget then tomorrow you’re reminded*.” (Participant 8).

Furthermore, the stakeholders suggested that delegating the training to a few selected, capable peers would be an effective way of implementing the programme and ensuring sustainability. “*My opinion is that few people can be trained who can act as ambassadors and train their colleagues at their respective places … this will facilitate training and ownership.*” (Participant 3)*.*

#### Availability of first aid training materials and equipment

Stakeholders highlighted the importance of utilizing materials and equipment that are familiar and easily available. They said this would enhance the possibility of providing care in the actual situation where sophisticated materials and equipment may not be available. “*I have seen on the TV they use plastic board to carry people … but can we easily have them here? I see that may be a challenge, so let us use what is available.*” (Participant 12). Furthermore, stakeholders suggested that the programme should provide user-friendly printed materials in the form of booklets or leaflets after completion of training so that they would be able to refer to important issues covered during training. “*After training provide us with easy-to-follow materials that we can always carry … we always need to remind ourselves.*” (Participant 9).

## Discussion

In this study, we explored perspectives of stakeholders regarding potential facilitators of and barriers to implementation of a PFA training programme for laypersons. The discussion will focus on identified facilitators and barriers under each of the themes of: Motivation for engaging in training, Constrains for engaging in training and Training processes.

The results concerning motivation for engaging in training related to perceived benefits of first aid training and availability of incentives. Our results show that perceptions of the potential benefits of the PFA training programme play an important role in motivating participants to engage in training. The results are in accordance with previous literature on human resource training which indicates a positive relationship between trainees’ motivation to participate in training and the perceived benefits of training [13–15]. In order to stimulate potential participants’ involvement in the training programmes and in implementation of training, it is important that inherent benefits of the training programmes are clearly stipulated and communicated to participants before implementation of training.

Furthermore, our results show that provision of financial incentives to compensate for the time spent away from normal income-generating activities, especially for participants who are not in formal employment, may facilitate participation in the training programme. The findings confirm the importance, as demonstrated in the literature, of offering financial incentives to maximize participation and implementation of programmes [16, 17]. One study which aimed to identify factors integral to the design and implementation of community-based cardiopulmonary resuscitation (CPR) intervention programmes reported how learners attributed successful implementation of the programme to provision of financial incentives [18]. However, while provision of incentives can indeed encourage programme participation, incentives should be used judiciously so that they do not compromise programme sustainability.

The results indicate how the work place situation influences implementation of the training programme, for example with regard to availability of time to attend training and accessibility of place of training. Our results showed that the big challenge to implement training programme was lack of time to participate in training because of other work-related responsibilities. Previous literature has likewise identified this as a major barrier to participation in training [19, 20]. To address this problem, various strategies that will make training shorter and time efficient need to be explored during programme designing. One alternative is to use blended learning approach were video-based self-instruction and face to face learning can be coordinated. Equally important there is a need to involve leaders and employers at some stages to understand goals of training so that they may be in position to render necessary support to their members and employees to participate in the training.

In addition, our results point to the need for training to be conducted close to the participants’ workplace so as to increase accessibility of training programme to participants by reducing travel distance and related costs. Similar views have been expressed by community members in a previous study aimed to identify factors perceived as barriers to learn and perform CPR in low income settings [21]. These results may suggest the need for tailoring training appropriate to the needs and contexts of participants.

The results concerning the training process related to methods of training delivery and availability of training materials and equipment. Practical-oriented and experience-based training was described as the preferred method of skills training. The results are in line with a previous review on the factors that influence the transfer of training in disaster preparedness training and also a study on barriers to implementation of CPR training, which indicated learners’ preference for experience-based learning methods [22, 23]. The method therefore needs to focus on skill-based training for laypersons because it has been shown to be effective in imparting knowledge and skills and also in boosting participants’ self-confidence as they learn to perform new skills [22]. Furthermore our results pointed to the need of involving motivated trainees to train their colleagues in order to reach more participants and increase sense of ownership and sustainability of the programme. However, further studies need to be conducted to investigate effectiveness of this approach to training of laypersons.

Our results indicate the need to use training materials that are available and relevant to the participants’ context. This was similarly reflected in a Ugandan study on training lay first responders where materials such as cardboard boxes, sheets and tarpaulin were used for various training purposes [24]. Furthermore, the results indicate the need to provide participants with easy-to-learn and easy-to-carry reference materials as well as periodic refresher courses after completion of training to strengthen and update knowledge and skills gained. This implies that programmes should have a strategy in place for periodic evaluation and continuing education so as to maintain knowledge and skills levels.

Our results point out some differences between groups in terms of motivation and constrains for engaging in training. The results amplify the need for tailoring training as a strategy to address concerns and priorities specific to particular groups. This may improve participation in the training and expected outcomes. Previous studies have also indicated positive effects of tailoring education programmes according to the needs and contexts of learners [25–27].

### Implication for prehospital care practice

The study informs about perspectives of stakeholders regarding motivation and constrains in implementing first aid training. Important areas on training processes are also discussed. The study highlights important contextual issues that can be considered when planning first aid training to maximize training outcome and sustainability.

### Limitations

Purposive sampling of leaders of stakeholder organizations was done with the view of obtaining rich information on the topic. However, the sample size was fairly small (*n* = 12), which could limit in explaining the complex situation of prehospital care in Tanzania. Social desirability bias may also have influenced stakeholder responses although the stakeholders were encouraged to be free and truthful in their responses.

This study focused on police and drivers, further research needs to be conducted to obtain views of other stakeholders such as health professionals, government administrators and policy makers to obtain a comprehensive picture about facilitators and barriers to the implementation of PFA training programmes.

## Conclusion

This study highlights potential facilitators and barriers to implementing a PFA training programme for laypersons, from the perspectives of leaders from police department and associations of city bus drivers, taxi drivers, and motorcycle taxis. It is hoped that this may be useful information for other stakeholders, and may enable government-level leaders and persons higher up in the health service hierarchy to take action to meet WHO recommendations for emergency pre-hospital care.

## Supplementary information

**Additional file 1.** Interview guide.

## Data Availability

The dataset generated and/or analysed during the current study is not publicly available owing to the confidential nature of qualitative data, but is available from the corresponding author on reasonable request.

## Abbreviations

CPR: Cardiopulmonary resuscitation
INPACT: Injury Prevention and Care in Tanzania
IRB: Institutional Review Board
LMICs: Low and middle-income countries
MUHAS: Muhimbili University of Health and Allied Sciences
PFA: Post-crash first aid
RTI: Road traffic injury
SIDA: Swedish International Development Cooperation Agency
WHO: World Health Organization

## References

[1] World Health Organisation (2016). Post-crash response: Supporting those affected by road traffic crashes.

[2] World Health Organization (2005). Prehospital trauma care sytems.

[3] Khorasani-zavareh D, Khankeh HR (2009). Post-crash management of road traffic injury victims in Iran. Stakeholders’ Views on current barriers and potential facilitators. BMC Emerg Med.

[4] Kironji A, Hodkinson P, De Ramirez S, Anest T, Wallis L, Razzak J, et al. Identifying barriers for out of hospital emergency care in low and low-middle income countries : a systematic review. BMC Health Serv Res. 2018;18(1):291. 10.1186/s12913-018-3091-0 PMC5907770 29673360

[5] Callese TE, Richards CT, Shaw P, Schuetz SJ, Issa N, Paladino L (2014). Layperson trauma training in low- and middle-income countries: a review. J Surg Res.

[6] Murad MK, Larsen S, Husum H (2012). Prehospital trauma care reduces mortality. Ten-year results from a time-cohort and trauma audit study in Iraq. Scand J Trauma Resusc Emerg Med.

[7] Jayaraman S, Mabweijano JR, Lipnick MS, Cadwell N, Miyamoto J, Wangoda R (2009). First things first: effectiveness and scalability of a basis prehospital trauma care program for lay first-responders in Kampala, Uganda. PLoS One.

[8] Tanzania Police Force and National Bureau of Statistics. Crime and traffic incidents statistics report 2016. Dar es Salaam: Tanzania Police Force; 2017.

[9] Lukumay GG, Ndile ML, Outwater AH, Mkoka DA, Padyab M, Saveman B (2018). Provision of post-crash first aid by traffic police in Dar es Salaam , Tanzania : a cross-sectional survey. BMC Emerg Med.

[10] Tanzania National Bureau of Statistics. Tanzania total population by districts-regions 2016/2017. Dar es Salaam: National Bureau of Statistics; 2017.

[11] Braun V, Clarke V (2006). Using thematic analysis in psychology. Qual Res Psychol.

[12] ICT Services and System Development and Division of Epidemiology and Global Health (2013). OpenCode 3.4.

[13] Aguinis Herman, Kraiger Kurt (2009). Benefits of Training and Development for Individuals and Teams, Organizations, and Society. Annual Review of Psychology.

[14] Machin MA, Treloar C (2004). Predictors of motivation to learn when training is mandatory. Proceedings of the 39th Australian Psychological Society Annual Conference: Psychological Science in Action.

[15] Lukianova L (2016). Motivation factors of adult learning. New Educ Rev.

[16] Koffarnus MN, Wong CJ, Fingerhood M, Svikis DS, Bigelow GESK (2013). Monetary incentives to reinforce engagement and achievement in a job-skills training program for homeless, unemployed adults. Appl Behav Anal.

[17] Bhattacharyya K, Winch P, LeBan K, Tien M (2001). Community health worker incentives and disincentives: How they affect motivation, retention, and sustainability.

[18] King R, Heisler M, Sayre M, Colbert S, Bond-Zielinski C, Rabe M (2015). Identification of factors integral to designing community-based CPR interventions for high-risk neighborhood residents. Prehospital Emerg Care.

[19] Graham R, Mccoy M, Schultz A (2015). Strategies to improve cardiac arrest survival: a time to act.

[20] Hovdhaugen E, Opheim V (2018). Participation in adult education and training in countries with high and low participation rates: demand and barriers. Int J lifelong Educ.

[21] Sasson C, Haukoos JS, Bond C, Rabe M, Colbert SH, King R (2013). Barriers and facilitators to learning and performing cardiopulmonary resuscitation in neighborhoods with low bystander cardiopulmonary resuscitation prevalence and high rates of cardiac arrest in Columbus, OH. Circ Cardiovasc Qual Outcomes.

[22] Kureckova V, Gabrhel V, Zamecnik P, Rezac P, Zaoral A, Obl J. First aid as an important traffic safety factor – evaluation of the experience–based training. Eur Transp Res Rev. 2017;9(1):5.

[23] Nazli N, Sipon S, Zumrah AR, Abdullah S (2015). The Factors that influence the transfer of training in disaster preparedness training: A review. Procedia - Soc Behav Sci.

[24] Jayaraman S, Mabweijano JR, Lipnick MS, Caldwell N, Miyamoto J, Wangoda R (2009). Current patterns of prehospital trauma care in Kampala, Uganda and the feasibility of a lay-first-responder training program. World J Surg.

[25] Schapira Marilyn M., Swartz Sheila, Ganschow Pamela S., Jacobs Elizabeth A., Neuner Joan M., Walker Cindy M., Fletcher Kathlyn E. (2017). Tailoring Educational and Behavioral Interventions to Level of Health Literacy: A Systematic Review. MDM Policy & Practice.

[26] Dizon JMR, Grimmer-Somers K, Kumar S (2014). Effectiveness of the tailored evidence based practice training program for Filipino physical therapists: a randomized controlled trial. BMC Med Educ.

[27] Brevik Thea Beate, Laake Petter, Bjørkly Stål (2020). Effect of culturally tailored education on attendance at mammography and the Papanicolaou test. Health Services Research.

