# Supplementary material for: Implementing a layperson post-crash first aid training programme in Tanzania: a qualitative study of stakeholder perspectives
Source: BMC Public Health. 2020 May 24;20:750. doi: 10.1186/s12889-020-08692-8 (PMC7245810; doi:10.1186/s12889-020-08692-8)
Supplement: Supplementary file 1 — Additional file 1. Interview guide. [file 12889_2020_8692_MOESM1_ESM.pdf]

INTERVIEW GUIDE

Views of stakeholders on potential barriers and facilitators in implementing post-crash first aid training programme for laypersons

Introduction

Thank you for taking part in this interview, firstly I would like to inform you that Injury Prevention and Care Tanzania (INPACT) project, under the Muhimbili University of Health and Allied Science is planning to conduct training programme aimed to build knowledge and skills of non-medical persons to provide first aid to road traffic injured victims. The purpose of this interview is to understand your views concerning potential factors that may enable or hinder implementation of such a programme. Your views as a stakeholder will help in making sure that the training programme is well designed and successful implemented.

Interview questions

1. Tell me your views concerning current situation in the provision of first aid to road injured victims in the country.
2. Are there any potential factors that may enable implementation of first aid training programme to non-medical persons? Please tell me.
3. Tell me your views regarding potential factors that may hinder implementation of first aid training programme to non-medical persons
4. What is your opinion regarding the best way to implement first aid training programme to non-medical persons like you?
5. Thank you for your participation in the interview, do you have any comments or suggestions regarding the implementation of first aid training programme to non-medical persons in the country?

END
